# Optical Coherence Tomography Angiography Features and Flow-Based Classification of Retinal Artery Macroaneurysms

**DOI:** 10.3390/jcm14248686

**Published:** 2025-12-08

**Authors:** Mohamed Oshallah, Anastasios E. Sepetis, Antonio Valastro, Eslam Ahmed, Sara Vaz-Pereira, Luca Ventre, Gabriella De Salvo

**Affiliations:** 1 Ophthalmology Department, University Hospital Southampton, Tremona Road, Southampton SO16 6YD, UK; 2 Ophthalmology Department, Beauregard Hospital, Azienda USL della Valle d’Aosta, Via L. Vaccari 5, 11100 Aosta, Italy; 3 Department of Ophthalmology, Faculdade de Medicina, Universidade de Lisboa, 1649-004 Lisbon, Portugal; 4 Clincal and Experimental Sciences, University of Southampton, Southampton SO16 6YD, UK

**Keywords:** angiography, OCTA, FFA, macroaneurysm

## Abstract

**Objectives:** We propose a flow-signal-based classification of retinal artery macroaneurysms (RAMs) using Optical Coherence Tomography Angiography (OCTA) and compare the findings with fundus fluorescein angiography (FFA). **Methods:** A retrospective review of 49 RAM cases observed over 6 years (October 2017–March 2023) at a medical retina clinic at the University Hospital Southampton, UK. Electronic clinical records, FFA, and OCTA images (en face and B-scan) were reviewed to identify pathology and assess RAM flow profiles. **Results:** In total, 30 eyes from 30 patients were included. The mean age of the patients was 76 years (range 49–91), with 17 females and 13 males. All eyes underwent OCTA, enabling classification of RAMs into three flow signal types: high (9 eyes), low (10 eyes), and absent (9 eyes), while 2 eyes had haemorrhage-related artefacts. A subgroup of 13 eyes also underwent FFA, allowing direct comparison, which showed flow profiles similar to those of OCTA: high (4 eyes), low (6 eyes), and absent (2 eyes), with 1 ungradable case due to subretinal haemorrhage masking. A discrepancy in flow was observed in one case where FFA indicated flow, but OCTA did not. Despite this, FFA and OCTA generally agreed on the flow levels, with a Spearman correlation of r = 0.79 (*p* = 0.004). **Conclusions:** OCTA flow profiles were directly comparable to FFA. OCTA effectively identified different levels of blood flow signal behaviour in RAMs. The proposed flow-based RAM classification may aid in prognosis, treatment indications, follow-up, and safe repeat imaging in clinical practice without systemic risk to the patient.

## 1. Introduction

Retinal arterial macroaneurysms (RAMs) are characterised by rounded or fusiform outpouchings of retinal arterioles. RAMs usually develop at weak points in arteries, such as arteriolar bifurcations, arteriovenous crossing points, or areas of localised arterial wall injury, in individuals predisposed to atherosclerosis [1]. The supratemporal artery is the most frequently reported site of involvement [2].

First described in 1973, these vascular anomalies are commonly seen in patients, particularly elderly women with systemic vascular diseases such as hypertension and atherosclerosis [2,3,4,5]. The incidence has been reported as 1 in 4500 adults in the Beijing Eye Study [6] and 1 in 1500 adults in the Central India Eye and Medical Study [7]. The development of RAMs is believed to result from arteriolar remodelling following vascular occlusion [8].

Although typically unilateral, RAMs can cause a wide range of ocular issues, from asymptomatic cases to severe bleeding and exudation, which may result in long-term subretinal fibrosis [9,10]. Treatment is only recommended for patients with reduced visual acuity, as most RAMs resolve on their own. Other therapeutic options, aside from observation for managing RAMs and their complications, include direct photocoagulation, pneumatic displacement with or without tissue plasminogen activator, surgical removal of haemorrhage via pars plana vitrectomy, and photo-disruption of the internal limiting membrane (ILM) or the posterior hyaloid using neodymium: yttrium-aluminium-garnet (Nd:YAG) laser or thermal laser to release the extravasated blood in cases of pre-macular haemorrhage [11,12,13]. Intra-vitreal anti-vascular endothelial growth factors (anti-VEGF) treatment has also been trialled with mixed results in cases with RAMs and macular exudation [14,15].

While RAMs are often clinically apparent and may not routinely require fundus fluorescein angiography (FFA) for diagnosis, various imaging techniques are commonly used in their assessment and monitoring. Patients usually undergo optical coherence tomography (OCT) and FFA to evaluate structural and vascular changes [16,17,18]. However, optical coherence tomography angiography (OCTA) offers a quick, non-invasive alternative for visualising flow characteristics, complementing clinical examination and potentially reducing the need for dye-based imaging in some instances.

RAMs have been extensively studied in the era of FFA. However, the advent of a new generation of OCTA has enabled improved visualisation of vascular flow dynamics. It allows for safe, repeatable imaging of the same pathology without exposing patients to the risks associated with dye-based techniques [19]. Furthermore, OCTA has provided us with a better understanding of the natural course of RAMs.

OCTA is indeed a relatively new imaging technique. It differs from other invasive ocular angiographic examinations, such as FFA and indocyanine green angiography (ICGA), which measure plasma flow. In contrast, OCTA detects red blood cell movement non-invasively. Besides the clear advantages of OCTA- such as improved patient comfort, lower risk, and shorter acquisition times- it can also distinguish between different retinal layers and visualise the various capillary plexuses. This feature of OCTA enables unique three-dimensional reconstruction and visualisation of the retinal vasculature, as well as the isolation of specific retinal plexuses. Furthermore, its depth-resolved analysis capability enables investigation of blood flow at specific axial locations within the retina and choroid. This groundbreaking ability to examine blood flow within a 3d retinal image may lead to new insights in the management of RAMs.

**Figure 1:**
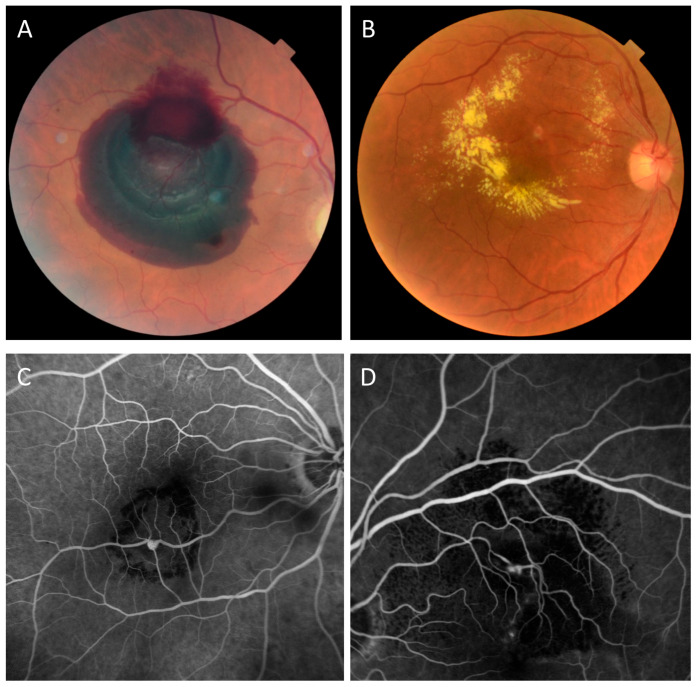
Multimodal imaging enables classification of retinal arterial macroaneurysms (RAMs) by functional type and morphology. (**A**) Colour fundus photograph of Type 1, hemorrhagic RAM. (**B**) Colour fundus photograph of Type 2, exudative RAM. (**C**) Fluorescein fundus angiogram (FFA), venous phase, RAM with saccular morphology. (**D**) FFA, venous phase, RAM with fusiform morphology.

The initial classification of RAMs was based on their hemorrhagic potential, dividing them into two groups: those that caused bleeds (Group 1, haemorrhagic) and those that caused exudates (Group 2, exudative) (Figure 1A,B) [5]. This was later expanded to include a quiescent type (non-haemorrhagic, non-exudative) [20]. Further details from angiographic studies enabled the classification of a second property of RAMs based on their appearance in angiography, categorising aneurysmal morphology as either fusiform or saccular (Figure 1C,D) [3]. RAMs are also classified by anatomical location, presence of visual symptoms, and whether aneurysmal decompensation is acute or chronic [20,21].

**Figure 2:**
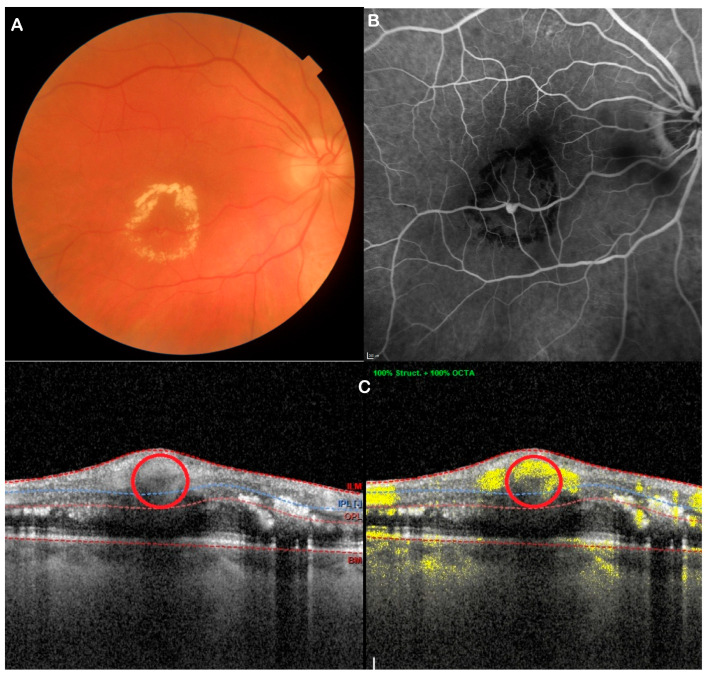
Multimodal imaging illustrates high-flow RAM. (**A**) Fundus photo; (**B**) FFA; (**C**) OCTA. **Red circle shows the RAM**.

**Figure 3:**
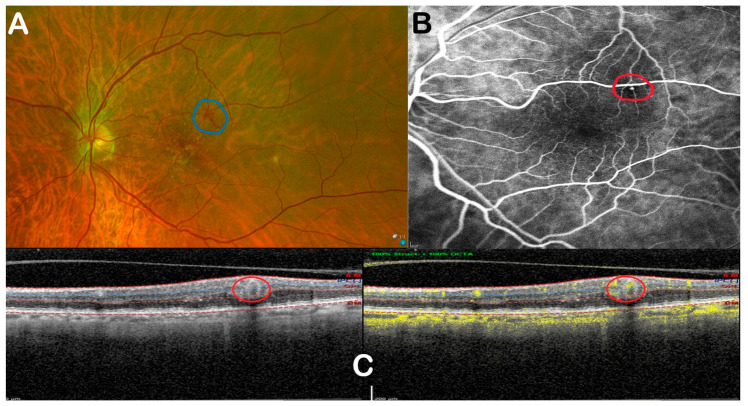
Multimodal imaging illustrates low-flow RAM. (**A**) Fundus photo; (**B**) FFA; (**C**) OCTA. **Red circle shows the RAM**.

**Figure 4:**
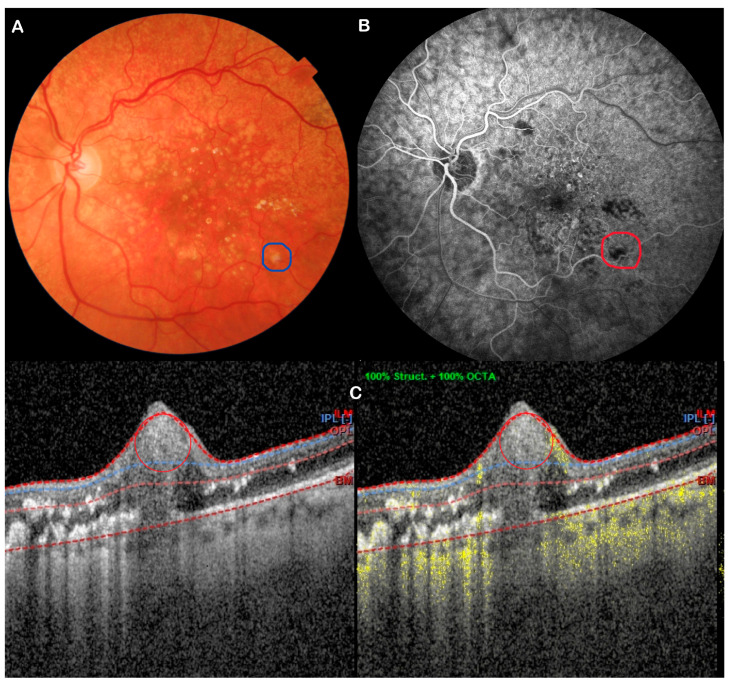
Multimodal imaging shows no-flow RAM. (**A**) Fundus photo; (**B**) FFA; (**C**) OCTA. **Red & blue circles show the RAM**.

OCTA imaging has not been systematically evaluated for its capacity to visualise microvascular details, detect flow within haemorrhagic RAMs, or distinguish perfused from non-perfused lesions. Furthermore, the comparative OCTA features of haemorrhagic versus non-haemorrhagic RAMs remain underreported, leaving uncertainty about the diagnostic value of flow-based assessments in these patients. By addressing these gaps, the present study aims to clarify the characteristic OCTA appearance of RAMs and to explore the potential of this modality to improve diagnostic accuracy and clinical understanding of these lesions. We therefore propose an OCTA-based classification of RAMs grounded on their flow signal (Figure 2, Figure 3 and Figure 4).

## 2. Materials and Methods

This is a single-centre, retrospective, observational study. Patients with RAMs attending a medical retina clinic were included over five years at University Hospital Southampton, UK. Forty-nine patients with RAMs were identified from electronic records between October 2017 and March 2023 (before transitioning to a different electronic system).

The study received approval from the Ethics Committee of the Eye Unit in Southampton (SEV/0766). It complied with the principles set out in the Declaration of Helsinki.

Inclusion criteria were patients aged 50 years or older with confirmed RAMs (abnormal retinal vessel structure, haemorrhage, or exudates) by two experienced ophthalmologists, who underwent OCTA with or without FFA on the same day.

**Scheme 1:**
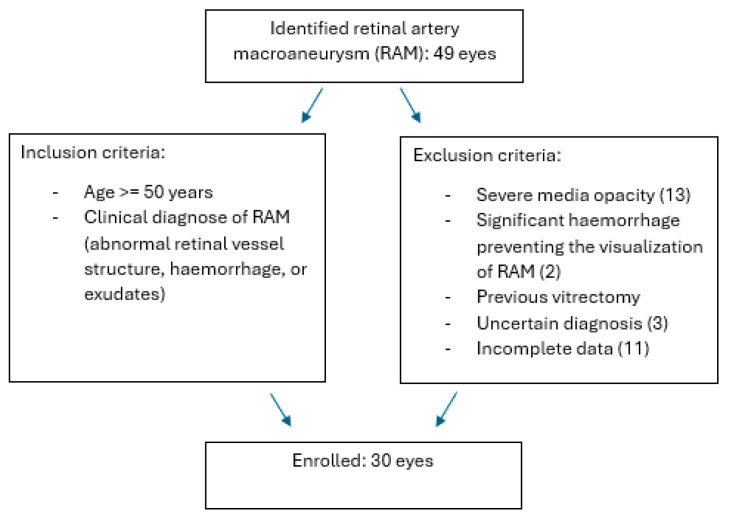
RAM (retinal artery macroaneurysm) inclusion and exclusion criteria.

Exclusion criteria included severe media opacity, significant haemorrhage preventing the visualisation of the RAM, previous vitrectomy, uncertain diagnoses, non-assessable imaging, and incomplete data. Patients with uncertain diagnoses, non-assessable imaging, and/or incomplete data were excluded (Scheme 1).

All patients included underwent a review of their medical records, a comprehensive ophthalmic examination, and OCTA with or without FFA using spectral domain-OCT (SD-OCT) Spectralis (Heidelberg Engineering, Heidelberg, Germany). Data were interpreted by two graders (EA and MO) who were masked to subject characteristics. In cases of disagreement, the final classification was determined through adjudication by a third senior grader (GDS), who reviewed the images and resolved the discrepancy by consensus. Inter-grader agreement before adjudication was assessed using linearly weighted Cohen’s kappa (weighted κ= 0.92, indicating good reliability).

OCTA was performed at presentation and, when available, during follow-up. The OCTA scans included a dense macular scan, 15-degree automatic reference scanning, and enhanced depth imaging (EDI) centred on the RAM lesion. Segmentation boundaries were manually corrected afterwards to ensure optimal visualization of the RAMs if segmentation artefacts occurred.

RAMs were classified based on various characteristics. Flow was recorded on FFA as early hyperfluorescence in the arterial phase, with well-defined filling that correlated with the RAM. If there was increasing hyperfluorescence in the mid-to-late phases, along with late leakage, the RAM was considered clinically active [16].

OCTA flow was assessed qualitatively on OCTA B-scan and en face images. High flow was defined as a visible flow signal occupying ≥ 50% of the aneurysmal lumen, low flow as <50%, and absent flow as minimal or no detectable intraluminal flow suggestive of thrombosis. Percentages were estimated visually by two senior graders (GDS, MO), as no quantitative lumen-flow algorithm is available in the current software.

Using OCTA, RAMs were classified as patent and active if a high-flow signal was present within the aneurysmal sac and if there was associated fluid, such as retinal thickening, oedema, or cysts, in the co-registered B-scan. If the flow signal was less than 50% of the RAM’s lumen, as estimated visually (a qualitative assessment commonly used in clinical settings), it was considered to have low flow. If the flow signal was absent or minimal (due to thrombosis) and little change was observed on the B-scan, the RAM was considered inactive and thrombosed and was classified as absent flow [22]. These characteristics were described in both the structural and transverse OCTA images. Additional features recorded included functional classification (haemorrhage, exudation, intraretinal fluid, and subretinal fluid) and morphology (saccular or fusiform). Furthermore, concomitant pathologies, including age-related macular degeneration, retinal vein occlusions, adult vitelliform macular dystrophy, and primary angle closure, were documented.

## 3. Results

After applying the exclusion criteria, 30 eyes from 30 patients with RAM were included from the original cohort of 49. Nineteen patients were excluded because neither FFA nor OCTA was performed. The mean age of the patients at baseline was 76 years (range 49–91), with 17 females and 13 males. Clinical and demographic characteristics are detailed in Table 1. Morphological and functional classifications are summarised in Table 2. Table 3 shows the characteristics of each case.

All eyes had OCTA, which allowed classification of RAMs into three different flow signal types: High (9 eyes) (Figure 2C), Low (10 eyes) (Figure 3C), and Absent (9 eyes) (Figure 3C), while 2 eyes exhibited blood-related artefacts. A subgroup of 13 eyes underwent FFA, enabling direct comparison, which showed similar flow profiles to OCTA: High (four eyes) (Figure 2B), Low (six eyes) (Figure 3B), and Absent flow (two eyes) (Figure 4B), with one ungradable case due to subretinal masking effects (Table 4). A discrepancy in flow was observed in one case: FFA showed flow, but OCTA showed no flow signal. Despite this, FFA and OCTA agreed on the level of flow, with a Spearman correlation of r = 0.79 (*p* = 0.004). Those with high flow displayed greater exudative changes.

## 4. Discussion

In our study, we observed results similar to previous research, indicating that RAMs are often found in elderly women and tend to be more common in individuals with uncontrolled systemic hypertension or arteriosclerosis [23]. Indeed, a significant proportion of our sample were female (17 out of 30 patients, 57%), with 21 patients having a history of hypertension and a mean age of 76 years. Although the ophthalmological assessment of RAMs involves clinical examination and various imaging techniques such as FFA, ICGA, and OCT, it is essential to perform a systemic risk factor assessment focused on arterial hypertension.

Sudden increases or fluctuations in systemic blood pressure can cause RAMs to rupture at weaker arterial points. This leads to blood dispersing throughout all retinal layers, sometimes even subretinally or into the vitreous gel, resulting in various clinical presentations. In some cases, RAMs may show exudation without haemorrhage due to an expanded vascular endothelial cell space. This exudation reduces the pressure within the RAM, thereby lowering the risk of rupture and subsequent haemorrhages [22].

RAMs can be classified as haemorrhagic, exudative, or quiescent, depending on whether subretinal/intraretinal fluid, hard exudates, or haemorrhages are present or absent. The purpose of this study was to classify RAMs based on flow factors observed on OCTA and to compare them with those observed on FFA (gold standard).

Sodium fluorescein is smaller and has a lower molecular weight than red blood cells, allowing it to permeate all regions of the RAM, including areas with intraluminal thrombi. Consequently, the morphological depiction of FFA closely resembles the actual anatomical structure of the RAM. In the initial FFA phase, the typical RAM fills uniformly, followed by a fusiform or saccular arteriolar dilation with possible delayed leakage. The presence of a thrombus within the RAM may lead to irregular filling. Hemorrhage may entirely or partially obscure the RAM’s visualisation with FFA. In such cases, ICGA is a valuable diagnostic alternative, as its absorption and emission peaks in the near-infrared spectrum enable deeper penetration through haemorrhagic areas. Furthermore, when using dynamic FFA, a pulsatile RAM can be observed in up to 10% of cases [1,3].

RAMs were categorised into three flow signal types on OCTA: high, low, and absent flow, with high-flow RAMs showing greater exudative changes. We used the 50% flow occupancy threshold as a practical and reproducible cut-off to distinguish between low- and high-flow RAM. While the assessment was mainly visual, supported by semi-quantitative image analysis, we believe this method reflects the real-world clinical setting, where precise pixel-based quantification is not always possible. Future studies using fully automated or quantitative algorithms may help refine this classification. Despite a single discrepancy, OCTA showed a strong correlation with FFA, underscoring its potential as a reliable, non-invasive imaging technique for RAMs.

Nevertheless, this examination carries risks; the main ones include severe respiratory reactions (1:3800), severe cardiac reactions (1:5300), and death (1:222,000) [24]. Furthermore, these risks seem higher in patients with significant respiratory and cardiac conditions. Since patients with RAMs often have multiple respiratory and cardiac comorbidities, there are usually relative contraindications to performing this examination.

OCTA, particularly swept-source OCTA (SS-OCTA), has been shown to provide a non-invasive method for tracking erythrocyte movement and pathways within RAM. Unlike traditional methods such as FFA, which can obscure anatomical details due to their smaller molecular weight and uniform distribution, SS-OCTA provides a clearer view of RAM morphology. This is especially useful for assessing turbulent flow or the presence of thrombi within lesions, as it traces the specific routes taken by red blood cells, reflecting the pathophysiological function of RAM. Furthermore, SS-OCTA reveals various appearances and sizes of RAM, indicating differences in flow dynamics and responses to treatment. Interestingly, some RAMs display distinct linear shapes, contrary to expected anatomical forms. Similarly, polyps, often considered cystic structures, can show linear features on SS-OCTA, challenging previous assumptions. Additionally, OCTA enables visualisation of RAM structures across different retinal layers, thereby enhancing three-dimensional localisation accuracy [22].

Various studies have emphasised the importance of OCTA in diagnosing and monitoring RAMs. Singh and Tripathy highlighted OCTA’s role in non-invasive imaging, improving diagnostic accuracy for RAMs without the risks tied to dye injections [25]. Goldhagen and Goldhardt reviewed various management strategies, emphasising OCTA’s usefulness in improving treatment outcomes [26]. OCTA’s ability to provide detailed, three-dimensional images of RAM morphology and flow dynamics has significant clinical implications. Vishal et al. demonstrated OCTA’s capacity to detect retinal racemose haemangioma and RAMs, showcasing its broad diagnostic potential [27]. Lokman et al. further confirmed OCTA’s role in the objective assessment of RAMs, supporting our study’s methodology and findings [28]. Our research also aligns with Brar et al., who demonstrated the utility of SS-OCTA in evaluating RAMs before and after combined laser and anti-VEGF therapy, providing insights into how treatments influence RAM morphology [29]. Another observational study of the OCTA appearance of RAMs [30] identified four vascular morphologic types of RAMs (i.e., distended, meshed, malformed, and occult), suggesting reproducible OCTA patterns. Yet, the absence of standardised operational definitions limits its clinical applicability. Additionally, Ouederni et al. discussed OCTA findings in IRVAN syndrome, including RAMs [31].

While this study provides a detailed description of RAM characteristics on OCTA, it does not investigate the relationship between flow profiles and clinical prognosis, treatment decisions, or long-term outcomes. Future longitudinal studies are needed to determine whether OCTA-based flow assessment can contribute to prognostic stratification or guide therapeutic planning.

Our study has additional limitations, as RAMs are often associated with cardiovascular diseases, such as severe hypertension, arteriosclerotic cardiovascular disease, and cardiac disease. During the retrospective review of cases, these conditions were considered relative contraindications to the procedure; therefore, some patients were either not suitable to proceed or declined to undergo an invasive diagnostic test. This exclusion may have introduced selection bias, potentially leaving the present cohort underrepresented in the broader RAM population. As such, the applicability of our findings to patients who cannot undergo FFA may be limited.

Furthermore, we found that none of our cases underwent ICGA. This is not unusual, as other studies report that FFA alone provides a more accurate representation of the actual anatomical structure due to sodium fluorescein’s smaller molecular weight, which allows for complete RAM filling [22].

The main advantage of our study, though, is the non-invasive classification of RAMs using OCTA, which enables detailed visualisation without the risks associated with traditional methods such as FFA. The classification described may have future relevance for the long-term management of patients with RAMs, as detailed OCTA assessment could help clinicians monitor lesion stability, detect perfusion changes, and identify cases that may benefit from closer observation or intervention. However, these implications remain preliminary given the study’s limitations. The relatively small sample size and retrospective design restrict generalisability, and the absence of longitudinal follow-up prevents assessment of whether specific flow features correlate with clinical outcomes or treatment responses. Prospective, multicentre studies are needed to validate the proposed flow-based classification, determine its prognostic utility, and evaluate whether OCTA-guided management strategies can optimise treatment selection and improve patient outcomes.

Looking ahead, automated OCTA analysis and machine-learning models could improve the reproducibility of flow assessment and enable the development of quantitative biomarkers for risk stratification. Future studies should also consider broader implementation factors—including cost-effectiveness, workflow efficiency, and patient acceptability—to fully evaluate the practicality of incorporating advanced OCTA-based classification into routine clinical care.

## Figures and Tables

**Table 1:** Demographics.

Demographics	
Males/Females (n)	13/17
Mean age (range)	76.0 (49–91)
Laterality Right/Left (n)	16/14
Eyes with OCTA (n)	30
Eyes with FFA (n)	13

**Table 2:** Morphological and functional classification.

	Functional Classification			Morphology			
	Type 1 Hemorrhagic	Type 2 Exudative	Ungradable	Saccular	Fusiform	Saccular & Fusiform	Ungradable
Total (n)	11	16	3	19	4	2	5
Percentage (%)	36.7%	53.3%	10%	63.3%	13.3%	6.7%	16.7%

**Table 3:** Flow characteristics (0: no flow, 1: low flow, 2: high flow) at OCTA (Optical Coherence Tomography Angiography) and FFA (Fundus Fluorescein Angiography), alongside demographics by case and associated copathologies (CRVO: Central Retinal Vein Occlusion; BRVO: Branch Retinal Vein Occlusion; AMD: Age-Related Macular Degeneration; AVMD: Adult Vitelliform Macular Dystrophy; PAC: Primary Angle Closure; DR: Diabetic Retinopathy; HTN-Ret: Hypertensive Retinopathy; Mac-Hole: Macular Hole; HCL: Hypercholesterolaemia; HTN: Hypertension).

	**Sex**	**Age**	**Copathology**	**HCL**	**HTN**	**OCTA Flow**	**FFA**	**FFA Flow**
1	M	72	CRVO	N	N	0	y	0
2	F	85	BRVO	N	Y	2	Y	2
3	F	91	AMD	N	N	2	Y	1
4	F	63	none	N	N	1	Y	1
5	M	83	AVMD	Y	Y	0	Y	1
6	F	74	none	N	N	2	Y	2
7	M	79	none	N	Y	2	Y	2
8	M	63	PAC	N	Y	2	Y	2
9	F	81	AMD	N	Y	Obscured	Y	Obscured
10	M	79	none	Y	Y	1	Y	1
11	F	84	none	N	Y	1	Y	0
12	M	69	DR	Y	Y	1	Y	1
13	F	75	none	N	Y	1	y	1
14	M	71	none	N	N	0	N	
15	F	60	HTN-Ret	N	Y	1	N	
16	F	88	AMD	N	Y	1	N	
17	M	74	DR	Y	N	1	N	
18	F	77	none	N	Y	2	N	
19	M	78	AMD	N	N	0	N	
20	M	79	none	Y	Y	2	N	
21	F	88	none	N	Y	0	N	
22	F	80	none	Y	Y	0	N	
23	F	76	Mac-Hole	N	Y	2	N	
24	M	49	none	N	N	0	N	
25	F	75	none	N	Y	0	N	
26	M	77	none	N	Y	0	N	
27	F	82	none	N	Y	1	N	
28	F	84	none	N	Y	2	N	
29	F	86	none	Y	Y	Obscured	N	
30	M	57	none	N	N	1	N	

**Table 4:** Flow distribution.

	Absent Flow (n)	Low Flow (n)	High Flow (n)	Blood Blocking (n)
OCTA	9	10	9	2
FFA	2	6	4	1

## Data Availability

The data that support the findings are available on reasonable request from the corresponding author, G.D.S.

## Abbreviations

The following abbreviations are used in this manuscript:

ARD EDI	Automatic reference scanning, Enhanced Depth Imaging
FFA	Fundus Fluorescein angiography
ICGA	Indocyanine Green Angiography
ILM	Internal Limiting membrane
OCT	Optical Coherence Tomography
OCTA	Optical Coherence Tomography Angiography
RAMs	Retinal artery macroaneurysms
SD-OCT	Spectral Domain-Optical Coherence Tomography
SS-OCTA	Swept Source-Optical Coherence Tomography Angiography
Nd:YAG	neodymium: yttrium-aluminum-garnet

## References

[1] Cahuzac A., Scemama C., Mauget-Faÿsse M., Sahel J.A., Wolff B. (2016). Retinal arterial macroaneurysms: Clinical, angiographic, and tomographic description and therapeutic management of a series of 14 cases. Eur. J. Ophthalmol..

[2] Pitkänen L., Tommila P., Kaarniranta K., Jääskeläinen J.E., Kinnunen K. (2014). Retinal arterial macroaneurysms. Acta Ophthalmol..

[3] Robertson D.M. (1973). Macroaneurysms of the retinal arteries. Trans. Am. Acad Ophthalmol. Otolaryngol..

[4] Cleary P.E., Kohner E.M., Hamilton A.M., Bird A.C. (1975). Retinal macroaneurysms. Br. J. Ophthalmol..

[5] Abdel-Khalek M.N., Richardson J. (1986). Retinal macroaneurysm: Natural history and guidelines for treatment. Br. J. Ophthalmol..

[6] Xu L., Wang Y., Jonas J.B. (2007). Frequency of retinal macroaneurysms in adult Chinese: The Beijing Eye Study. Br. J. Ophthalmol..

[7] Nangia V., Jonas J.B., Khare A., Sinha A., Lambat S. (2013). Prevalence of retinal macroaneurysms. The Central India Eye and Medical Study. Acta Ophthalmol..

[8] Panton R.W., Goldberg M.F., Farber M.D. (1990). Retinal arterial macroaneurysms: Risk factors and natural history. Br. J. Ophthalmol..

[9] Theodossiadis P.G., Emfietzoglou I., Sfikakis P.P., Panagiotidis D., Grigoropoulos V.G., Theodossiadis G.P. (2005). Simultaneous bilateral visual loss caused by rupture of retinal arterial macroaneurysms in a hypertensive patient. Acta Ophthalmol. Scand..

[10] Lewis R.A., Norton E.W., Gass J.D. (1976). Acquired arterial macroaneurysms of the retina. Br. J. Ophthalmol..

[11] Gedik S., Gür S., Yilmaz G., Akova Y.A. (2007). Retinal arterial macroaneurysm rupture following fundus fluorescein angiography and treatment with Nd: YAG laser membranectomy. Ophthalmic Surg. Lasers Imaging.

[12] Hillenkamp J., Surguch V., Framme C., Gabel V.P., Sachs H.G. (2010). Management of submacular hemorrhage with intravitreal versus subretinal injection of recombinant tissue plasminogen activator. Graefes Arch. Clin. Exp. Ophthalmol..

[13] Parodi M.B., Iacono P., Ravalico G., Bandello F. (2011). Subthreshold laser treatment for retinal arterial macroaneurysm. Br. J. Ophthalmol..

[14] Pichi F., Morara M., Torrazza C., Manzi G., Alkabes M., Balducci N., Vitale L., Lembo A., Ciardella A.P., Nucci P. (2013). Intravitreal bevacizumab for macular complications from retinal arterial macroaneurysms. Am. J. Ophthalmol..

[15] Cho W.H., Chiang W.Y., Chen C.H., Kuo H.K. (2020). To treat or not to treat: A clinical series of retinal arterial macroaneurysms: A single-center retrospective study. Medicine.

[16] Moosavi R.A., Fong K.C.S., Chopdar A. (2006). Retinal artery macroaneurysms: Clinical and fluorescein angiographic features in 34 patients. Eye.

[17] Goldenberg D., Soiberman U., Loewenstein A., Goldstein M. (2012). Heidelberg spectral-domain optical coherence tomographic findings in retinal artery macroaneurysm. Retina.

[18] Miura M., Muramatsu D., Hong Y.J., Yasuno Y., Itami A., Iwasaki T., Goto H. (2015). Noninvasive vascular imaging of ruptured retinal arterial macroaneurysms by Doppler optical coherence tomography. BMC Ophthalmol..

[19] Breazzano M.P., Fernández-Avellaneda P., Freund K.B. (2019). Swept-Source Optical Coherence Tomography Angiography of Retinal Arterial Macroaneurysm With Overlying Hemorrhage. JAMA Ophthalmol..

[20] Lavin M.J., Marsh R.J., Peart S., Rehman A. (1987). Retinal arterial macroaneurysms: A retrospective study of 40 patients. Br. J. Ophthalmol..

[21] Palestine A.G., Robertson D.M., Goldstein B.G. (1982). Macroaneurysms of the retinal arteries. Am. J. Ophthalmol..

[22] Song Y., Zhang W., Yu S., Gong Y. (2023). Appearance of retinal arterial macroaneurysms in patients using swept-source optical coherence tomographic angiography. BMC Ophthalmol..

[23] Rabb M.F., Gagliano D.A., Teske M.P. (1988). Retinal arterial macroaneurysms. Surv. Ophthalmol..

[24] Yannuzzi L.A., Rohrer K.T., Tindel L.J., Sobel R.S., Costanza M.A., Shields W., Zang E. (1986). Fluorescein angiography complication survey. Ophthalmology.

[25] Singh D., Tripathy K. (2025). Retinal Macroaneurysm. StatPearls [Internet].

[26] Evan Goldhagen B., Goldhardt R. (2019). Retinal Arterial Macroaneurysms: Updating your Memory on RAM Management. Curr. Ophthalmol. Rep..

[27] Vishal R., Avadesh O., Srinivas R., Taraprasad D. (2018). Retinal racemose hemangioma with retinal artery macroaneurysm: Optical coherence tomography angiography (OCTA) findings. Am. J. Ophthalmol Case Rep..

[28] Mohd Lokman M., Catherine Bastion M.L., Che Hamzah J. (2022). Objective Assessment of Retinal Artery Macroaneurysm With Optical Coherence Tomography Angiography. Cureus.

[29] Brar M., Grewal S.P.S., Grewal D.S., Sharma M., Dogra M.R. (2022). Swept source optical coherence tomography angiography of a case of retinal artery macro-aneurysm before and after combined laser and intra-vitreal ranibizumab treatment. Indian J. Ophthalmol..

[30] Hu L., Zhang L., Li Z., Liu J., Yu R., Wang L., Xing D., Li C., Yang Y., Li X. (2023). Features of Vasculature in Patients with Retina Arterial Macroaneurysm Using Optical Coherence Tomography Angiography. Retina.

[31] Ouederni M., Sassi H., Chelly Z., Nefaa F., Cheour M. (2022). Optical Coherence Tomography Angiography in idiopathic retinal vasculitis, aneurysms and neuroretinitis (IRVAN) syndrome: A case report. Eur. J. Ophthalmol..

